# Role of p53 in the Regulation of the Inflammatory Tumor Microenvironment and Tumor Suppression

**DOI:** 10.3390/cancers10070219

**Published:** 2018-06-27

**Authors:** Ikuno Uehara, Nobuyuki Tanaka

**Affiliations:** Department of Molecular Oncology, Institute for Advanced Medical Sciences, Nippon Medical School, 1-396 Kosugi-cho, Nakahara-ku, Kawasaki 211-8533, Japan; uehar@nms.ac.jp

**Keywords:** p53, inflammation, tumor microenvironment, cancer stem cells, nuclear factor-κB (NF-κB), cancer metabolism, oxidative stress, cellular reprograming

## Abstract

p53 has functional roles in tumor suppression as a guardian of the genome, surveillant of oncogenic cell transformation, and as recently demonstrated, a regulator of intracellular metabolism. Accumulating evidence has shown that the tumor microenvironment, accompanied by inflammation and tissue remodeling, is important for cancer proliferation, metastasis, and maintenance of cancer stem cells (CSCs) that self-renew and generate the diverse cells comprising the tumor. Furthermore, p53 has been demonstrated to inhibit inflammatory responses, and functional loss of p53 causes excessive inflammatory reactions. Moreover, the generation and maintenance of CSCs are supported by the inflammatory tumor microenvironment. Considering that the functions of p53 inhibit reprogramming of somatic cells to stem cells, p53 may have a major role in the inflammatory microenvironment as a tumor suppressor. Here, we review our current understanding of the mechanisms underlying the roles of p53 in regulation of the inflammatory microenvironment, tumor microenvironment, and tumor suppression.

## 1. Introduction

Increasing evidence has indicated that oncogenesis is a multistep process that reflects genetic alterations driving the progressive transformation of normal cells into highly malignant derivatives [1,2]. Such genetic alterations produce oncogenes with a dominant gain of function and tumor suppressor genes with a recessive loss of function. *p53* is the most frequently mutated gene in human cancer cells [3]. It functions as a transcriptional activator and exerts its biological activity by inducing the expression of its target genes [4]. p53 is regulated by the ubiquitin E3 ligase Mdm2 that targets p53 for proteasomal degradation. In response to various cellular stresses, such as DNA damage, hypoxia, and nutrient deprivation, p53 induces the expression of genes regulating the cell cycle, apoptosis, and DNA repair [4,5,6]. Through these functions, p53 prevents passing on altered genetic information evoked by DNA damage and has therefore been termed the “guardian of the genome” [7,8]. Moreover, the p53-mediated tumor surveillance system, which responds to oncogenic signals evoked by oncogene activation, triggers apoptosis or cell cycle arrest, resulting in the elimination of oncogene-activated cells by cell death or induction of senescence [5,7,9]. Therefore, inactivation of this system by the loss of p53 functions is a critical step in cancer development. Moreover, p53 responds to metabolic changes and influences metabolic pathways through several mechanisms. The metabolic alterations induced by p53 mutations might play a major role in the maintenance of cancers [9,10]. For example, p53 promotes oxidative phosphorylation and hampers glycolysis in cells, and disruption of this balance is critical for oncogene-induced cell transformation [11,12]. In addition, mice bearing mutations in three p53 acetylation sites, which cause deficiencies in cell cycle arrest, senescence, and apoptosis, retain tumor suppression activity [13]. This mutant p53 retains the ability to regulate energy metabolism, suggesting that, in addition to regulation of the cell cycle and apoptosis, regulation of metabolism is an important function of p53-mediated tumor suppression.

Most tumors have missense mutations that generate an aberrant p53 protein [14]. Growing evidence has shown that these mutant p53 proteins have lost wild-type p53 tumor suppressor activity and gained functions that contribute to malignant progression [15]. For example, a p53 missense mutation in the germline results in significantly earlier cancer onset than loss of p53 protein expression [16,17], and mice expressing mutant p53 develop more aggressive and metastatic tumors than p53 null mice [18,19]. Moreover, many types of p53 mutants in cancer cells confer resistance to anti-cancer drugs [15,20]. Although many different mutations and various phenotypes exist in tumors expressing different p53 mutants, the mechanisms underlying oncogenic functions of p53 mutants are not fully understood [15]. Identification of small molecules that restore the wild-type conformation of p53 and its proper functions is progressing [21].

Recent studies have demonstrated the roles of p53 in regulation of the inflammatory tumor microenvironment and cell fate reprograming. Therefore, this review will focus on the roles of p53 in regulation of the inflammatory tumor microenvironment, including the generation and maintenance of cancer stem cells (CSCs) and tumor suppression.

## 2. The Inflammatory Microenvironment and Its Effects on Cancer Cells

Tumor-promoting inflammation is one of the hallmarks of cancer [2]. Chronic inflammatory disease increases the risk of cancers. Epidemiological evidence shows that anti-inflammatory drugs, particularly aspirin, are powerful chemopreventive agents [22]. Furthermore, tumorigenesis and wound healing depend on similar molecular mechanisms [23,24]. Wound-healing responses initiate a cascade of events including immune cell migration to the wound site to prevent infection and remove debris, followed by fibroblast proliferation, remodeling of the extracellular matrix, angiogenesis, and the deposition of new connective tissue [23,25]. Initially, inflammatory cells, including neutrophils, mast cells, macrophages, and lymphocytes, migrate to the wound site and produce growth factors, cytokines, and proteinases required for new tissue formation. These inflammatory mediators facilitate the recruitment, proliferation, and differentiation of mesenchymal stem cells (MSCs) that alter the local inflammatory microenvironment and facilitate the wound repair activities of intrinsic tissue stem cells by producing immunoregulatory factors, growth factors, and chemokines [26]. Inflammatory cells also migrate to cancer tissues, and MSCs have been reported to migrate to tumors and differentiate into cells such as tumor-associated MSCs (TA-MSCs) [27]. Similar to the wound-healing microenvironment, in the tumor microenvironment, inflammatory cells, TA-MSCs, cancer associated fibroblasts (CAFs), and cancer cells themselves produce inflammatory cytokines, such as interleukin (IL)-6, IL-1β, and tumor necrosis factor-α (TNF-α, chemokines, such as MCP1 (monocyte chemoattractant protein 1) and IL-8, and growth factors including members of the EGF (epidermal growth factor), FGF (fibroblast growth factor), and transforming growth factor β (TGF-β families, all of which enhance tumor development [23,28]. Furthermore, the extracellular matrix (ECM) interacts with cells to regulate diverse functions including proliferation, migration, and differentiation. Dysregulation of the ECM composition and structure caused by inflammation contributes to several pathological conditions including cancer invasion and metastasis [29,30]. Moreover, additional cell growth signals induced by growth factors [31] and inflammatory cytokines [32,33,34] enhance the energy metabolism of cancer cells in the tumor microenvironment. This tumor microenvironment contributes to evasion of anti-tumor immune responses [35]. Based on these findings, inhibition of tumor microenvironment components might provide a more efficient method of cancer chemotherapy [36].

Epithelial-mesenchymal transition (EMT) is a biological process whereby a polarized epithelial cell, which normally interacts with the basement membrane via its basal surface, undergoes multiple biochemical changes to assume a mesenchymal cell phenotype characterized by an enhanced migratory capacity, invasiveness, and increased resistance to apoptosis [37]. Furthermore, inflammatory mediators, including proinflammatory cytokines, growth factors, oxidative stress, and toxins, induce EMT that promotes cancer progression [38]. Epithelial-mesenchymal transition is regulated by EMT-inducing transcription factors such as Twist1/2, Snail1/2, and Zeb1/2 [39]. In addition, wild-type p53 interacts with Snail2 to promote Snail2 degradation, whereas p53 mutations upregulates Twist1 expression to enhance Snail2 expression by inhibiting its degradation [40,41], suggesting a connection between p53 inhibition and EMT. Moreover, EMT promotes the generation of CSCs from differentiated cancer cells [42].

Like normal stem cells, CSCs reside in microenvironments called niches that regulate stem cell fate by providing signals from cell-cell contacts and secreted factors. Niches for mammalian stem cells have been identified in various epithelial tissues such as the intestinal, neural, epidermal, and hematopoietic systems [43]. CSC niches consist of immune cells, stromal cells, such as TA-MSCs and CAFs, and their secreted factors including growth factors and cytokines that stimulate CSC self-renewal. Because many cancer cells have an aberrant differentiation status, multiple developmental signals can be activated in CSCs and niches. Such developmental signals, including HEDGEHOG (HH), WNT, and NOTCH, may facilitate maintenance of CSC stemness and regulation of their self-renewal [44]. Moreover, CSCs show increased quiescence and poor responses to conventional chemotherapeutic strategies that primarily kill proliferating cells [45]. These mechanisms contribute to the survival of CSCs during drug treatment, leading to tumor recurrence [46]. Therefore, stem cell-based therapies may be a promising strategy for cancer chemotherapy, especially by inhibiting tumor recurrence. 

## 3. p53-Mediated Regulation of Inflammation

In addition to the role of p53 in maintaining cellular homeostasis in response to various stresses, accumulating evidence suggests the involvement of p53 in inflammatory reactions [47,48]. Cancers arise from sites of chronic inflammation [49,50], and p53 is induced by chronic inflammation through various stimuli including oxidative stress initiated by reactive oxygen species (ROS) [51], nitric oxide (NO) [52], and viral [53] and bacterial [54] infections. Considering these findings and the tumor-suppressive function of p53, p53 functions might be suppressed at sites of inflammation. Numerous studies have reported that the transcription factor nuclear factor-κB (NF-κB) is a key regulator linking persistent infections and chronic inflammation to increased cancer risk [50]. Indeed, NF-κB inhibits the transcriptional activity of p53 [55,56,57]. Moreover, in several renal cell carcinoma cell lines, inhibition of NF-κB restores p53 functions [58]. In this context, curaxins, small molecules that simultaneously inhibit NF-κB and activate p53, have shown anti-cancer activities against human tumor xenografts in mice [59].

Moreover, p53 suppresses inflammation [60]. For example, a significant number of p53-null mice die before tumor development from inflammation resulting in abscesses, gastroenteritis, or myocarditis [61]. Furthermore, symptoms of autoimmune disease are more severe in p53-deficient mice than in wild-type mice [62,63,64]. However, inflammation caused by loss of p53 itself is not sufficient for effective tumor development. In a mouse model of carcinogen-induced colon cancer, intestinal stem cell marker Lgr5-driven deletion of p53 reduces apoptosis and increases proliferation of crypt stem cells but has no effect on tumor incidence or size. Conversely, in a mouse model of colitis-associated cancer, stem cell-specific p53 deletion greatly enhances tumor size and incidence in the colon, suggesting that the loss of p53 functions in stem cells enables colonic tumor formation only when combined with DNA damage and chronic inflammation [65]. One of the mechanisms involved in enhanced tumor development of p53-deficient mice might be suppression of NF-κB by p53 [47,60]. In addition to the suppression of p53 by NF-κB, p53 and NF-κB compete for the limited transcription coactivator p300 and the closely related cyclic adenosine monophosphate (cAMP) response element-binding protein-binding protein (CBP), resulting in their mutual inhibition [55,56,57]. Furthermore, we have found that p53 directly suppresses transcriptional activity of the NF-κB subunit p65 through inhibition of p65 binding and p65 activation by IκB kinase (IKK) β [66]. In addition, p53 inhibits NF-κB by suppressing proteasomal degradation of IκB (an inhibitor of NF-κB) [67]. Considering these findings, p53 might function as a restrictor and attenuator of inflammatory reactions via the balance between p53 and NF-κB.

## 4. Role of p53 in the Inflammatory Tumor Microenvironment

In addition to the reciprocal inhibitory mechanism between p53 and NF-κB-mediated inflammation, studies have reported the role of p53 in the inflammatory tumor microenvironment [60,68]. Cancer-associated mutant p53, whose transcriptional activity is lost, augments NF-κB transcriptional activity in response to the cytokine TNF-α. Conversely, downregulation of the mutant p53 sensitizes cancer cells to the apoptotic effects of TNF-α [69]. Mutant p53 promotes inflammatory responses by repression of secreted interleukin-1 receptor antagonist (sIL-1Ra) [70]. Moreover, p53 mutants enhance TLR3 (Toll-like-receptor 3; a pattern recognition receptor for pathogens)-induced cytokine and chemokine expression in response to its ligand [71]. The role of mutant p53 has also been analyzed in the regulation of gene expression [68,72]. It has been recently shown that p53 mutants interact directly with NF-κB, and that TNF-α signaling promotes the enhancer binding and transcriptional interplay between mutant p53 and NF-κB [73]. These findings suggest that, in the inflammatory tumor microenvironment, cancer cells that harbor a p53 mutation enhance inflammation in response to inflammatory cytokines/chemokines and pathogens. Therefore, this positive feedback activation system in cancer cells and their microenvironment may enhance tumor development using the chronic inflammation-tissue regeneration system described above.

Senescence induced in oncogene-expressing cells is a p53-dependent tumor-suppressor mechanism that prevents malignant transformation by suppressing cellular proliferation [5]. Senescence is also characterized by secretion of a set of cytokines and chemokines known as the senescence-associated secretory phenotype (SASP) by constitutively active NF-κB [74]. However, NF-κB acts as a master regulator of SASP, and NF-κB suppression cooperates with p53 inactivation to bypass senescence, suggesting dual roles of NF-κB [74]. In relation to this finding, the ability of NF-κB under some circumstances to behave as a tumor suppressor has also been discussed [75]. From another viewpoint, it is possible that acquisition of a SASP phenotype also changes senescent fibroblasts into proinflammatory cells that promote tumor progression [76]. Moreover, it has been reported that mutant p53 prolongs NF-κB activation and promotes chronic inflammation and inflammation-associated cancer [30]. In addition to oncogene-expressing cells, p53 is induced in aging cells through various mechanisms including the accumulation of oxidative damage triggered by ROS [75]. Therefore, in aging tissues or tissues that acquire oncogenic DNA changes, such as continuous exposure to mutagens or infection by oncogenic viruses, p53-induced senescence followed by senescence-induced production of cytokines/chemokines in the tissue microenvironment might induce chronic inflammation and enhance the risk of cancer.

## 5. Metabolic Changes Caused by p53 in the Inflammatory Tumor Microenvironment

The loss of p53 functions changes the source of energy from cellular respiration to glycolysis. This metabolic shift towards aerobic glycolysis, termed the Warburg effect, has been observed in cancer cells [10,76]. We previously found that IKKα/β activity, the transcriptional activity of NF-κB, and glycolysis are enhanced in *p53*-deficient cells, and that oncogenic Ras-induced cell transformation and enhanced aerobic glycolysis in *p53*-deficient cells depend on NF-κB [11]. Moreover, we reported that enhanced glycolysis-induced O-GlcNAcylation of IKKβ in *p53*-deficient cells activates NF-κB, which subsequently induces glucose transporter 3 (GLUT3) expression. This observation suggests that the positive feedback activation from enhanced glucose metabolism to NF-κB-induced GLUT3 expression is involved in the enhanced energy production of cancer cells [77]. Moreover, p53 regulates mitochondrial respiration by synthesis of cytochrome c oxidase 2 (SCO2), a cytochrome c oxidase assembly factor, and loss of p53 inhibits oxygen consumption [12]. Therefore, the loss of p53 functions may induce a Warburg effect-type metabolic shift using these mechanisms. In cancer tissues, the regulation of energy production systems by cellular interactions is complex. Lactate from a hypoxic, glycolytic tumor cell population fuels ATP production in the oxygenated region of a tumor, indicating that lactate is an important metabolite for oxidative phosphorylation in neighboring oxygenated cancer cells and tissues. Furthermore, this metabolic symbiosis, the so-called “reverse Warburg effect”, induces aerobic glycolysis of CAFs and provides cancer cells with metabolites for oxidative phosphorylation [78,79,80]. In addition, under hypoxic conditions in the cancer microenvironment, the transcription factor hypoxia inducible factor (HIF)-1 induces GLUT1, glycolytic enzymes, and pyruvate dehydrogenase kinase-1 (PDK-1) that limit pyruvate utilization for oxidative phosphorylation and enhance aerobic glycolysis [81,82]. Moreover, growth factors [31] and inflammatory cytokines [32,33,34] enhance the energy metabolism of cancer cells, suggesting that cytokines promote metabolic symbiosis with surrounding cancer stromal cells in combination with suppression of p53 by NF-κB.

Reactive oxygen species are produced as byproducts of mitochondrial respiration, other cellular elements, exogenous pollutants, pathogens, and radiation. They modulate various cell signaling pathways that are mediated by transcription factors, including NF-κB, signal transducer and activator of transcription 3 (STAT3), and HIF-1, which are linked to inflammation, tumor growth, invasion, and metastasis [83,84]. p53 is induced by oxidative stress initiated by ROS [51], which monitors and maintains ROS levels to prevent excessive ROS accumulation that might compromise genome integrity [85]. Indeed, p53 functions as a regulator of antioxidant genes that restrict ROS levels and prevent endogenous oxidative stress [86,87]. In this context, p53 is activated by NO, a free radical, produced in the colon tissues of patients with ulcerative colitis, a cancer-prone inflammatory bowel disease, and activated p53 protects against NO-induced DNA damage [88]. Therefore, in the inflammatory tumor microenvironment, p53 may be a negative feedback regulator of oxidative stress, which suppresses oxidative stress-induced tumor promoting events. 

## 6. Maintenance of CSCs in the Tumor Microenvironment and p53

In 1863, Rudolf Virchow, “the father of modern pathology”, noted leucocytes in neoplastic tissues and proposed a connection between inflammation and cancer. He suggested that the “lymphoreticular infiltrate” reflected the origin of cancer at sites of chronic inflammation [24]. Crosstalk between cancer development and inflammatory processes was proved experimentally, and its underlying mechanism has been analyzed at the molecular level [49,50]. Moreover, recent findings have suggested that, similar to induced pluripotent stem (iPS) cells, oncogenic genomic mutations under an inflammatory microenvironment promote cancer development through chromatin remodeling caused by epigenetic plasticity [89,90]. In relation to this notion, it was stated that “tumors are wounds that do not heal” [91]. The wound-healing process triggered by inflammation is followed by new tissue formation and tissue remodeling. In the tumor microenvironment, stromal cells in wounds and tumors, including fibroblasts, endothelial cells, and inflammatory cells, are important regulators of the migration and proliferation of normal epithelial cells in wounds and cancer cells in tumors [23,25]. In addition, although the effects on tissue repair and regeneration by multiple activated metabolic pathways are not fully understood, activation of metabolic pathways including glycolysis has been observed in injured tissues, especially in infiltrating macrophages [92]. In the inflammatory tumor microenvironment, inflammatory and stromal cells are considered to constitute niches that regulate stem cell fate by providing signals in the form of cell-cell contacts and secreted factors [43]. Therefore, it has been suggested that targeting the tumor microenvironment may useful for chemoprevention of cancer [26,93]. Because genetic analysis of mammary tumor stromal cells obtained by laser capture microdissection has revealed a high frequency of mutations in the *p53* gene [94], it is also important to consider suppression and mutation of p53 for such treatment. Moreover, inflammation promotes selection of cells with oncogenic mutations including p53 mutations [95,96]. Thus, inflammation can select for oncogenic mutations, and the growing oncogenic clone would promote further inflammation as a feed-forward loop to enhance tumor development [95,96].

Studies have shown that p53 inhibits reprogramming of somatic cells to stem cells [97]. iPS cells produced by enforced expression of genes encoding four transcription factors, c-Myc, Klf4, Sox2, and Oct3/4, in mouse fibroblasts have the same capabilities as embryonic stem cells, including self-renewal and differentiation into all tissue types [98]. During the production of iPS cells, inactivation of p53 markedly increases the efficiency [99,100,101,102]. Moreover, p53 deficiency has simplified iPS cells generation by introducing only two factors, Oct3/4 and Sox2 [103]. In salamanders, a group of amphibians, p53 activity is critical for limb regeneration [104]. This finding suggests that p53 is critical for controlling key cell fate decisions throughout regulation of dedifferentiation and redifferentiation events, and that deregulation of these events by mutation of p53 might result in cancer development. MSCs are adult stem cells that differentiate into various cell types of mesodermal origin, including bone, cartilage, fat, muscle and marrow stroma [105]. Furthermore, p53 regulates the proliferation and differentiation of MSCs [106]. These findings suggest that p53 regulates the differentiation of stem cells and suppresses dedifferentiation. Therefore, the loss of functional p53 supports dedifferentiation, maintains the stemness of cells, and induces the generation of CSCs. Moreover, reprogramming factors Oct3/4, Sox2, Klf4, and c-Myc evoke senescence and reprogramming. Under expressing these factors, p53 limits in vivo reprogramming, and its inhibitor, Ink4a/Arf, promotes in vivo reprogramming through the production of IL-6 that creates a permissive tissue microenvironment for in vivo reprogramming [107]. Therefore, biological conditions linked to senescence, such as tissue injury and aging, favor in vivo reprogramming.

In the inflammatory tumor microenvironment, various mechanisms suppress the functions of p53 as described in Section 3 and Section 4. Previous studies have reported the role of inflammation in the maintenance and generation of CSCs [43,108]. For example, the critical role of STAT3 induced by IL-6 and other inflammatory cytokines has been widely reported for the maintenance of CSCs [109]. IL-8, also known as CXCL8 (C-X-C motif chemokine ligand 8), is a potent neutrophil attractant and activator produced by monocytes and macrophages in response to inflammation. Interleukin-8 and its receptors, C-X-C motif chemokine receptor (CXCR) 1/CXCR2, induce the maintenance of CSCs [110,111]. Moreover, developmental signals, such as HH, WNT, and NOTCH, are produced in the tumor microenvironment and might maintain the stemness of CSCs and regulate their self-renewal [44,112]. In this context, these signals inhibit p53 [113,114,115]. Furthermore, CSCs exhibit an altered energy balance and metabolic status such as enhanced glycolysis compared with non-CSC counterparts in various tumor types. The distinct metabolic phenotype of CSCs is similar to the metabolic phenotype of normal stem cells, suggesting the importance of the metabolic shift to support stemness properties [116,117]. In addition, O-GlcNAcylation regulates the reprogramming of somatic cells to iPS cells and their pluripotency through modification of the core reprogramming factors Oct3/4 and Sox2 [118]. O-GlcNAcylation is promoted by the hexosamine biosynthetic pathway that is enhanced by increased glucose flux in response to increased glycolysis and has important roles in cancer-relevant processes such as cell signaling, transcription, cell division, metabolism, and cytoskeletal regulation [119]. Therefore, in the tumor microenvironment, inflammation-induced metabolic changes might be involved in the generation of CSCs and oncogenesis.

## 7. Conclusions

**Figure 1:**
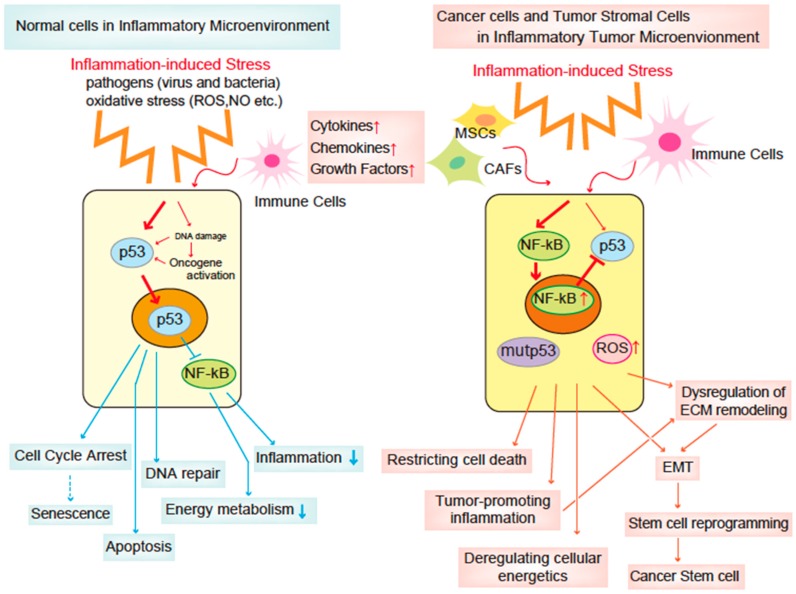
Role and regulatory mechanism of p53 in the inflammatory tumor microenvironment. p53 is regulated by inflammation and inhibits the generation and maintenance of cancer stem cells (CSCs). In the absence of p53 functions, enhanced inflammatory reactions cause cells to be continuously exposed to inflammatory cytokines, chemokines, and growth factors, resulting in accumulation of DNA damage induced by oxidative stress and enhanced energy metabolism. These conditions might cause reprogramming of the cells to facilitate the production of CSCs, tumor development, and metastasis.

Accumulating studies have demonstrated the roles and regulatory mechanisms of p53 in the inflammatory tumor microenvironment. Based on the evidence described above, the relationship between p53 and inflammation in cancer can be considered as follows (Figure 1). In response to inflammation induced by infections, wounds or tissue damage, p53 is activated to suppress inflammation. Under these conditions, inflammation also restricts p53 functions by several mechanisms including NF-κB. In addition to p53 regulating this fine-tuning system of inflammation, p53 hampers energy metabolism. These regulatory systems of p53 in inflammation and metabolism inhibit the generation and maintenance of CSCs via a p53-mediated barrier system that inhibits reprogramming of somatic cells to undifferentiated stem cells. Conversely, in the absence of p53 functions, enhanced inflammatory reactions cause cells in the local microenvironment to be continuously exposed to inflammatory cytokines, chemokines, and growth factors, resulting in accumulation of DNA damage induced by oxidative stress and enhanced energy metabolism. These conditions might cause reprogramming of cells to facilitate the production of CSCs, tumor development, and metastasis. In this context, p53 might function as a cellular guardian by reducing the oncogenic effects of chronic inflammation. Therefore, it is desirable to develop a therapeutic such as TA-MSCs, CAFs, and inflammatory cells, and the reprogramming system that induces CSCs and that converts mutant p53 to the wild-type phenotype. It has also been considered that NF-κB activation in chronic inflammation is involved in cancer development of Barrett’s esophagus as a precursor of esophageal adenocarcinoma, *Helicobacter pylori–*induced gastric cancer, and colon cancers [120]. Such a role of NF-κB has also been demonstrated in experimental animal models [50]. Furthermore, tumor stromal cells in the inflammatory microenvironment are considered to be a target for cancer therapy [26,93]. Considering these findings and the abovementioned role of p53, it is conceivable that the reciprocal inhibition and relationship between NF-κB and p53 are important regulatory mechanisms of oncogenesis in the inflammatory tumor microenvironment.

Recently, small molecules that reactivate missense mutant p53 and induce tumor cell death have been identified by various approaches [20,21]. Indeed, CP-31398 was identified as a compound that protects wild-type p53 from thermal denaturation and restores wild-type functions of some p53 mutants. Moreover, PRIMA-1 and APR-246 promote refolding of mutant p53 and induce p53 target genes. A growing number of small molecules that promote proper folding and/or reactivation of missense-mutant p53 proteins have been identified. Among these compounds, several have significant antitumor activities in cultured tumor cells and various mouse tumor models [20,21]. In addition to the compounds that restore wild-type activity to p53 mutants, the compounds that selectively promote mutant p53 degradation are expected to be candidate therapeutic agents [15]. Considering the deficiency in tumor suppressor functions of p53 and the effect of the cancer-promoting activity of mutant p53 in cancer cells, it is conceivable that p53-targeted therapy could effectively act on cancer cells themselves and the cancer microenvironment.

## References

[1] Hanahan D., Weinberg R.A. (2000). The hallmarks of cancer. Cell.

[2] Hanahan D., Weinberg R.A. (2011). Hallmarks of cancer: The next generation. Cell.

[3] Hamroun D., Kato S., Ishioka C., Claustres M., Beroud C., Soussi T. (2006). The UMD TP53 database and website: Update and revisions. Hum. Mutat..

[4] Vogelstein B., Lane D., Levine A.J. (2000). Surfing the p53 network. Nature.

[5] Lowe S.W., Cepero E., Evan G. (2004). Intrinsic tumour suppression. Nature.

[6] Oren M. (2003). Decision making by p53: Life, death and cancer. Cell. Death. Differ..

[7] Efeyan A., Serrano M. (2007). p53: Guardian of the genome and policeman of the oncogenes. Cell Cycle.

[8] Lane D.P. (1992). Cancer. p53, guardian of the genome. Nature.

[9] Levine A.J., Oren M. (2009). The first 30 years of p53: Growing ever more complex. Nat. Rev. Cancer.

[10] Vousden K.H., Ryan K.M. (2009). p53 and metabolism. Nat. Rev. Cancer.

[11] Kawauchi K., Araki K., Tobiume K., Tanaka N. (2008). p53 regulates glucose metabolism through an IKK-NF-kappaB pathway and inhibits cell transformation. Nat. Cell. Biol..

[12] Matoba S., Kang J.G., Patino W.D., Wragg A., Boehm M., Gavrilova O., Hurley P.J., Bunz F., Hwang P.M. (2006). p53 regulates mitochondrial respiration. Science.

[13] Li T., Kon N., Jiang L., Tan M., Ludwig T., Zhao Y., Baer R., Gu W. (2012). Tumor suppression in the absence of p53-mediated cell-cycle arrest, apoptosis, and senescence. Cell.

[14] Kandoth C., McLellan M.D., Vandin F., Ye K., Niu B., Lu C., Xie M., Zhang Q., McMichael J.F., Wyczalkowski M.A. (2013). Mutational landscape and significance across 12 major cancer types. Nature.

[15] Muller P.A., Vousden K.H. (2014). Mutant p53 in cancer: New functions and therapeutic opportunities. Cancer Cell.

[16] Bougeard G., Sesboue R., Baert-Desurmont S., Vasseur S., Martin C., Tinat J., Brugieres L., Chompret A., de Paillerets B.B., Stoppa-Lyonnet D. (2008). Molecular basis of the Li-Fraumeni syndrome: An update from the French LFS families. J. Med. Genet..

[17] Zerdoumi Y., Aury-Landas J., Bonaiti-Pellie C., Derambure C., Sesboue R., Renaux-Petel M., Frebourg T., Bougeard G., Flaman J.M. (2013). Drastic effect of germline TP53 missense mutations in Li-Fraumeni patients. Hum. Mutat..

[18] Lang G.A., Iwakuma T., Suh Y.A., Liu G., Rao V.A., Parant J.M., Valentin-Vega Y.A., Terzian T., Caldwell L.C., Strong L.C. (2004). Gain of function of a p53 hot spot mutation in a mouse model of Li-Fraumeni syndrome. Cell.

[19] Olive K.P., Tuveson D.A., Ruhe Z.C., Yin B., Willis N.A., Bronson R.T., Crowley D., Jacks T. (2004). Mutant p53 gain of function in two mouse models of Li-Fraumeni syndrome. Cell.

[20] Hientz K., Mohr A., Bhakta-Guha D., Efferth T. (2017). The role of p53 in cancer drug resistance and targeted chemotherapy. Oncotarget.

[21] Bykov V.J.N., Eriksson S.E., Bianchi J., Wiman K.G. (2018). Targeting mutant p53 for efficient cancer therapy. Nat. Rev. Cancer.

[22] Crusz S.M., Balkwill F.R. (2015). Inflammation and cancer: Advances and new agents. Nat. Rev. Clin. Oncol..

[23] Arwert E.N., Hoste E., Watt F.M. (2012). Epithelial stem cells, wound healing and cancer. Nat. Rev. Cancer.

[24] Balkwill F., Mantovani A. (2001). Inflammation and cancer: Back to Virchow?. Lancet.

[25] Schafer M., Werner S. (2008). Cancer as an overhealing wound: An old hypothesis revisited. Nat. Rev. Mol. Cell. Biol..

[26] Shi Y., Du L., Lin L., Wang Y. (2017). Tumour-associated mesenchymal stem/stromal cells: Emerging therapeutic targets. Nat. Rev. Drug. Discov..

[27] Quante M., Tu S.P., Tomita H., Gonda T., Wang S.S., Takashi S., Baik G.H., Shibata W., Diprete B., Betz K.S. (2011). Bone marrow-derived myofibroblasts contribute to the mesenchymal stem cell niche and promote tumor growth. Cancer Cell.

[28] Pedersen T.X., Leethanakul C., Patel V., Mitola D., Lund L.R., Dano K., Johnsen M., Gutkind J.S., Bugge T.H. (2003). Laser capture microdissection-based in vivo genomic profiling of wound keratinocytes identifies similarities and differences to squamous cell carcinoma. Oncogene.

[29] Bonnans C., Chou J., Werb Z. (2014). Remodelling the extracellular matrix in development and disease. Nat. Rev. Mol. Cell. Biol..

[30] Oudin M.J., Jonas O., Kosciuk T., Broye L.C., Guido B.C., Wyckoff J., Riquelme D., Lamar J.M., Asokan S.B., Whittaker C. (2016). Tumor Cell-Driven Extracellular Matrix Remodeling Drives Haptotaxis during Metastatic Progression. Cancer Discov..

[31] Vander Heiden M.G., Cantley L.C., Thompson C.B. (2009). Understanding the Warburg effect: The metabolic requirements of cell proliferation. Science.

[32] Ando M., Uehara I., Kogure K., Asano Y., Nakajima W., Abe Y., Kawauchi K., Tanaka N. (2010). Interleukin 6 enhances glycolysis through expression of the glycolytic enzymes hexokinase 2 and 6-phosphofructo-2-kinase/fructose-2,6-bisphosphatase-3. J. Nippon. Med. Sch..

[33] Ben-Shlomo I., Kol S., Roeder L.M., Resnick C.E., Hurwitz A., Payne D.W., Adashi E.Y. (1997). Interleukin (IL)-1beta increases glucose uptake and induces glycolysis in aerobically cultured rat ovarian cells: Evidence that IL-1beta may mediate the gonadotropin-induced midcycle metabolic shift. Endocrinology.

[34] Vaughan R.A., Garcia-Smith R., Trujillo K.A., Bisoffi M. (2013). Tumor necrosis factor alpha increases aerobic glycolysis and reduces oxidative metabolism in prostate epithelial cells. Prostate.

[35] Spranger S., Gajewski T.F. (2018). Impact of oncogenic pathways on evasion of antitumour immune responses. Nat. Rev. Cancer.

[36] Belli C., Trapani D., Viale G., D'Amico P., Duso B.A., Della Vigna P., Orsi F., Curigliano G. (2018). Targeting the microenvironment in solid tumors. Cancer Treat. Rev..

[37] Brabletz T., Kalluri R., Nieto M.A., Weinberg R.A. (2018). EMT in cancer. Nat. Rev. Cancer.

[38] Perez L., Munoz-Durango N., Riedel C.A., Echeverria C., Kalergis A.M., Cabello-Verrugio C., Simon F. (2017). Endothelial-to-mesenchymal transition: Cytokine-mediated pathways that determine endothelial fibrosis under inflammatory conditions. Cytokine Growth Factor Rev..

[39] Puisieux A., Brabletz T., Caramel J. (2014). Oncogenic roles of EMT-inducing transcription factors. Nat. Cell Biol..

[40] Kogan-Sakin I., Tabach Y., Buganim Y., Molchadsky A., Solomon H., Madar S., Kamer I., Stambolsky P., Shelly A., Goldfinger N. (2011). Mutant p53(R175H) upregulates Twist1 expression and promotes epithelial-mesenchymal transition in immortalized prostate cells. Cell Death Differ..

[41] Wang S.P., Wang W.L., Chang Y.L., Wu C.T., Chao Y.C., Kao S.H., Yuan A., Lin C.W., Yang S.C., Chan W.K. (2009). p53 controls cancer cell invasion by inducing the MDM2-mediated degradation of Slug. Nat. Cell. Biol..

[42] Lamouille S., Xu J., Derynck R. (2014). Molecular mechanisms of epithelial-mesenchymal transition. Nat. Rev. Mol. Cell Biol..

[43] Plaks V., Kong N., Werb Z. (2015). The cancer stem cell niche: How essential is the niche in regulating stemness of tumor cells?. Cell Stem Cell.

[44] Reya T., Morrison S.J., Clarke M.F., Weissman I.L. (2001). Stem cells, cancer, and cancer stem cells. Nature.

[45] Colak S., Medema J.P. (2014). Cancer stem cells--important players in tumor therapy resistance. FEBS J..

[46] Dean M., Fojo T., Bates S. (2005). Tumour stem cells and drug resistance. Nat. Rev. Cancer.

[47] Cooks T., Harris C.C., Oren M. (2014). Caught in the cross fire: p53 in inflammation. Carcinogenesis.

[48] Gudkov A.V., Gurova K.V., Komarova E.A. (2011). Inflammation and p53: A Tale of Two Stresses. Genes Cancer.

[49] Elinav E., Nowarski R., Thaiss C.A., Hu B., Jin C., Flavell R.A. (2013). Inflammation-induced cancer: Crosstalk between tumours, immune cells and microorganisms. Nat. Rev. Cancer.

[50] Taniguchi K., Karin M. (2018). NF-kappaB, inflammation, immunity and cancer: Coming of age. Nat. Rev. Immunol..

[51] Reuter S., Gupta S.C., Chaturvedi M.M., Aggarwal B.B. (2010). Oxidative stress, inflammation, and cancer: How are they linked?. Free Radic. Biol. Med..

[52] Goodman J.E., Hofseth L.J., Hussain S.P., Harris C.C. (2004). Nitric oxide and p53 in cancer-prone chronic inflammation and oxyradical overload disease. Environ. Mol. Mutagen..

[53] Rivas C., Aaronson S.A., Munoz-Fontela C. (2010). Dual Role of p53 in Innate Antiviral Immunity. Viruses.

[54] Siegl C., Rudel T. (2015). Modulation of p53 during bacterial infections. Nat. Rev. Microbiol..

[55] Ikeda A., Sun X., Li Y., Zhang Y., Eckner R., Doi T.S., Takahashi T., Obata Y., Yoshioka K., Yamamoto K. (2000). p300/CBP-dependent and -independent transcriptional interference between NF-kB RelA and p53. Biochem. Biophys. Res. Commun..

[56] Wadgaonkar R., Phelps K.M., Haque Z., Williams A.J., Silverman E.S., Collins T. (1999). CREB-binding protein is a nuclear integrator of nuclear factor-kB and p53 signaling. J. Biol. Chem..

[57] Webster G.A., Perkins N.D. (1999). Transcriptional cross talk between NF-kB and p53. Mol. Cell. Biol..

[58] Gurova K.V., Hill J.E., Guo C., Prokvolit A., Burdelya L.G., Samoylova E., Khodyakova A.V., Ganapathi R., Ganapathi M., Tararova N.D. (2005). Small molecules that reactivate p53 in renal cell carcinoma reveal a NF-kappaB-dependent mechanism of p53 suppression in tumors. Proc. Natl. Acad. Sci. USA.

[59] Gasparian A.V., Burkhart C.A., Purmal A.A., Brodsky L., Pal M., Saranadasa M., Bosykh D.A., Commane M., Guryanova O.A., Pal S. (2011). Curaxins: Anticancer compounds that simultaneously suppress NF-kappaB and activate p53 by targeting FACT. Sci. Transl. Med..

[60] Gudkov A.V., Komarova E.A. (2016). p53 and the Carcinogenicity of Chronic Inflammation. Cold Spring Harb. Perspect. Med..

[61] Donehower L.A., Harvey M., Slagle B.L., McArthur M.J., Montgomery C.A., Butel J.S., Bradley A. (1992). Mice deficient for p53 are developmentally normal but susceptible to spontaneous tumours. Nature.

[62] Okuda Y., Okuda M., Bernard C.C. (2003). Regulatory role of p53 in experimental autoimmune encephalomyelitis. J. Neuroimmunol..

[63] Yamanishi Y., Boyle D.L., Pinkoski M.J., Mahboubi A., Lin T., Han Z., Zvaifler N.J., Green D.R., Firestein G.S. (2002). Regulation of joint destruction and inflammation by p53 in collagen-induced arthritis. Am. J. Pathol..

[64] Zheng S.J., Lamhamedi-Cherradi S.E., Wang P., Xu L., Chen Y.H. (2005). Tumor suppressor p53 inhibits autoimmune inflammation and macrophage function. Diabetes.

[65] Davidson L.A., Callaway E.S., Kim E., Weeks B.R., Fan Y.Y., Allred C.D., Chapkin R.S. (2015). Targeted Deletion of p53 in Lgr5-Expressing Intestinal Stem Cells Promotes Colon Tumorigenesis in a Preclinical Model of Colitis-Associated Cancer. Cancer Res..

[66] Kawauchi K., Araki K., Tobiume K., Tanaka N. (2008). Activated p53 induces NF-kappaB DNA binding but suppresses its transcriptional activation. Biochem. Biophys. Res. Commun..

[67] Son D.S., Kabir S.M., Dong Y.L., Lee E., Adunyah S.E. (2012). Inhibitory effect of tumor suppressor p53 on proinflammatory chemokine expression in ovarian cancer cells by reducing proteasomal degradation of IkappaB. PLoS ONE.

[68] Cordani M., Pacchiana R., Butera G., D’Orazi G., Scarpa A., Donadelli M. (2016). Mutant p53 proteins alter cancer cell secretome and tumour microenvironment: Involvement in cancer invasion and metastasis. Cancer Lett..

[69] Weisz L., Damalas A., Liontos M., Karakaidos P., Fontemaggi G., Maor-Aloni R., Kalis M., Levrero M., Strano S., Gorgoulis V.G. (2007). Mutant p53 enhances nuclear factor kappaB activation by tumor necrosis factor alpha in cancer cells. Cancer Res..

[70] Ubertini V., Norelli G., D’Arcangelo D., Gurtner A., Cesareo E., Baldari S., Gentileschi M.P., Piaggio G., Nistico P., Soddu S. (2015). Mutant p53 gains new function in promoting inflammatory signals by repression of the secreted interleukin-1 receptor antagonist. Oncogene.

[71] Menendez D., Lowe J.M., Snipe J., Resnick M.A. (2016). Ligand dependent restoration of human TLR3 signaling and death in p53 mutant cells. Oncotarget.

[72] Weisz L., Oren M., Rotter V. (2007). Transcription regulation by mutant p53. Oncogene.

[73] Rahnamoun H., Lu H., Duttke S.H., Benner C., Glass C.K., Lauberth S.M. (2017). Mutant p53 shapes the enhancer landscape of cancer cells in response to chronic immune signaling. Nat. Commun..

[74] Campisi J., Robert L. (2014). Cell senescence: Role in aging and age-related diseases. Interdiscip. Top. Gerontol..

[75] Rufini A., Tucci P., Celardo I., Melino G. (2013). Senescence and aging: The critical roles of p53. Oncogene.

[76] Koppenol W.H., Bounds P.L., Dang C.V. (2011). Otto Warburg's contributions to current concepts of cancer metabolism. Nat. Rev. Cancer.

[77] Kawauchi K., Araki K., Tobiume K., Tanaka N. (2009). Loss of p53 enhances catalytic activity of IKKβ through O-linked β-N-acetyl glucosamine modification. Proc. Natl. Acad. Sci. USA.

[78] Bonuccelli G., Tsirigos A., Whitaker-Menezes D., Pavlides S., Pestell R.G., Chiavarina B., Frank P.G., Flomenberg N., Howell A., Martinez-Outschoorn U.E. (2010). Ketones and lactate “fuel” tumor growth and metastasis: Evidence that epithelial cancer cells use oxidative mitochondrial metabolism. Cell Cycle.

[79] Migneco G., Whitaker-Menezes D., Chiavarina B., Castello-Cros R., Pavlides S., Pestell R.G., Fatatis A., Flomenberg N., Tsirigos A., Howell A. (2010). Glycolytic cancer associated fibroblasts promote breast cancer tumor growth, without a measurable increase in angiogenesis: Evidence for stromal-epithelial metabolic coupling. Cell Cycle.

[80] Nakajima E.C., Van Houten B. (2013). Metabolic symbiosis in cancer: Refocusing the Warburg lens. Mol. Carcinog..

[81] Denko N.C. (2008). Hypoxia, HIF1 and glucose metabolism in the solid tumour. Nat. Rev. Cancer.

[82] Keith B., Johnson R.S., Simon M.C. (2011). HIF1α and HIF2α: Sibling rivalry in hypoxic tumour growth and progression. Nat. Rev. Cancer.

[83] Prasad S., Gupta S.C., Tyagi A.K. (2017). Reactive oxygen species (ROS) and cancer: Role of antioxidative nutraceuticals. Cancer Lett..

[84] Sabharwal S.S., Schumacker P.T. (2014). Mitochondrial ROS in cancer: initiators, amplifiers or an Achilles’ heel?. Nat. Rev. Cancer.

[85] Liu B., Chen Y., St Clair D.K. (2008). ROS and p53: A versatile partnership. Free Radic. Biol. Med..

[86] Budanov A.V., Sablina A.A., Feinstein E., Koonin E.V., Chumakov P.M. (2004). Regeneration of peroxiredoxins by p53-regulated sestrins, homologs of bacterial AhpD. Science.

[87] Polyak K., Xia Y., Zweier J.L., Kinzler K.W., Vogelstein B. (1997). A model for p53-induced apoptosis. Nature.

[88] Hofseth L.J., Saito S., Hussain S.P., Espey M.G., Miranda K.M., Araki Y., Jhappan C., Higashimoto Y., He P., Linke S.P. (2003). Nitric oxide-induced cellular stress and p53 activation in chronic inflammation. Proc. Natl. Acad. Sci. USA.

[89] Buschbeck M., Hake S.B. (2017). Variants of core histones and their roles in cell fate decisions, development and cancer. Nat. Rev. Mol. Cell. Biol..

[90] Giroux V., Rustgi A.K. (2017). Metaplasia: Tissue injury adaptation and a precursor to the dysplasia-cancer sequence. Nat. Rev. Cancer.

[91] Dvorak H.F. (1986). Tumors: Wounds that do not heal. Similarities between tumor stroma generation and wound healing. N. Engl. J. Med..

[92] Eming S.A., Wynn T.A., Martin P. (2017). Inflammation and metabolism in tissue repair and regeneration. Science.

[93] Albini A., Sporn M.B. (2007). The tumour microenvironment as a target for chemoprevention. Nat. Rev. Cancer.

[94] Kurose K., Gilley K., Matsumoto S., Watson P.H., Zhou X.P., Eng C. (2002). Frequent somatic mutations in PTEN and TP53 are mutually exclusive in the stroma of breast carcinomas. Nat. Genet..

[95] Henry C.J., Casas-Selves M., Kim J., Zaberezhnyy V., Aghili L., Daniel A.E., Jimenez L., Azam T., McNamee E.N., Clambey E.T. (2015). Aging-associated inflammation promotes selection for adaptive oncogenic events in B cell progenitors. J. Clin. Invest..

[96] Vermeulen L., Morrissey E., van der Heijden M., Nicholson A.M., Sottoriva A., Buczacki S., Kemp R., Tavare S., Winton D.J. (2013). Defining stem cell dynamics in models of intestinal tumor initiation. Science.

[97] Krizhanovsky V., Lowe S.W. (2009). Stem cells: The promises and perils of p53. Nature.

[98] Takahashi K., Yamanaka S. (2006). Induction of pluripotent stem cells from mouse embryonic and adult fibroblast cultures by defined factors. Cell.

[99] Hong H., Takahashi K., Ichisaka T., Aoi T., Kanagawa O., Nakagawa M., Okita K., Yamanaka S. (2009). Suppression of induced pluripotent stem cell generation by the p53-p21 pathway. Nature.

[100] Li H., Collado M., Villasante A., Strati K., Ortega S., Canamero M., Blasco M.A., Serrano M. (2009). The Ink4/Arf locus is a barrier for iPS cell reprogramming. Nature.

[101] Marion R.M., Strati K., Li H., Murga M., Blanco R., Ortega S., Fernandez-Capetillo O., Serrano M., Blasco M.A. (2009). A p53-mediated DNA damage response limits reprogramming to ensure iPS cell genomic integrity. Nature.

[102] Utikal J., Polo J.M., Stadtfeld M., Maherali N., Kulalert W., Walsh R.M., Khalil A., Rheinwald J.G., Hochedlinger K. (2009). Immortalization eliminates a roadblock during cellular reprogramming into iPS cells. Nature.

[103] Kawamura T., Suzuki J., Wang Y.V., Menendez S., Morera L.B., Raya A., Wahl G.M., Izpisua Belmonte J.C. (2009). Linking the p53 tumour suppressor pathway to somatic cell reprogramming. Nature.

[104] Yun M.H., Gates P.B., Brockes J.P. (2013). Regulation of p53 is critical for vertebrate limb regeneration. Proc. Natl. Acad. Sci. USA.

[105] Pittenger M.F., Mackay A.M., Beck S.C., Jaiswal R.K., Douglas R., Mosca J.D., Moorman M.A., Simonetti D.W., Craig S., Marshak D.R. (1999). Multilineage potential of adult human mesenchymal stem cells. Science.

[106] Armesilla-Diaz A., Elvira G., Silva A. (2009). p53 regulates the proliferation, differentiation and spontaneous transformation of mesenchymal stem cells. Exp. Cell Res..

[107] Mosteiro L., Pantoja C., Alcazar N., Marion R.M., Chondronasiou D., Rovira M., Fernandez-Marcos P.J., Munoz-Martin M., Blanco-Aparicio C., Pastor J. (2016). Tissue damage and senescence provide critical signals for cellular reprogramming in vivo. Science.

[108] Sica A., Porta C., Amadori A., Pasto A. (2017). Tumor-associated myeloid cells as guiding forces of cancer cell stemness. Cancer. Immunol. Immunother..

[109] Yu H., Lee H., Herrmann A., Buettner R., Jove R. (2014). Revisiting STAT3 signalling in cancer: New and unexpected biological functions. Nat. Rev. Cancer.

[110] Ginestier C., Liu S., Diebel M.E., Korkaya H., Luo M., Brown M., Wicinski J., Cabaud O., Charafe-Jauffret E., Birnbaum D. (2010). CXCR1 blockade selectively targets human breast cancer stem cells in vitro and in xenografts. J. Clin. Invest..

[111] Hwang W.L., Yang M.H., Tsai M.L., Lan H.Y., Su S.H., Chang S.C., Teng H.W., Yang S.H., Lan Y.T., Chiou S.H. (2011). SNAIL regulates interleukin-8 expression, stem cell-like activity, and tumorigenicity of human colorectal carcinoma cells. Gastroenterology.

[112] Takebe N., Miele L., Harris P.J., Jeong W., Bando H., Kahn M., Yang S.X., Ivy S.P. (2015). Targeting Notch, Hedgehog, and Wnt pathways in cancer stem cells: clinical update. Nat. Rev. Clin. Oncol..

[113] Abe Y., Oda-Sato E., Tobiume K., Kawauchi K., Taya Y., Okamoto K., Oren M., Tanaka N. (2008). Hedgehog signaling overrides p53-mediated tumor suppression by activating Mdm2. Proc. Natl. Acad. Sci. USA.

[114] Beverly L.J., Felsher D.W., Capobianco A.J. (2005). Suppression of p53 by Notch in lymphomagenesis: Implications for initiation and regression. Cancer Res..

[115] Riascos-Bernal D.F., Chinnasamy P., Cao L.L., Dunaway C.M., Valenta T., Basler K., Sibinga N.E. (2016). β-Catenin C-terminal signals suppress p53 and are essential for artery formation. Nat. Commun..

[116] Folmes C.D., Nelson T.J., Martinez-Fernandez A., Arrell D.K., Lindor J.Z., Dzeja P.P., Ikeda Y., Perez-Terzic C., Terzic A. (2011). Somatic oxidative bioenergetics transitions into pluripotency-dependent glycolysis to facilitate nuclear reprogramming. Cell. Metab..

[117] Wong T.L., Che N., Ma S. (2017). Reprogramming of central carbon metabolism in cancer stem cells. Biochim Biophys Acta.

[118] Jang H., Kim T.W., Yoon S., Choi S.Y., Kang T.W., Kim S.Y., Kwon Y.W., Cho E.J., Youn H.D. (2012). O-GlcNAc regulates pluripotency and reprogramming by directly acting on core components of the pluripotency network. Cell Stem Cell.

[119] Slawson C., Hart G.W. (2011). O-GlcNAc signalling: Implications for cancer cell biology. Nat. Rev. Cancer.

[120] Merga Y.J., O'Hara A., Burkitt M.D., Duckworth C.A., Probert C.S., Campbell B.J., Pritchard D.M. (2016). Importance of the alternative NF-κB activation pathway in inflammation-associated gastrointestinal carcinogenesis. Am. J. Physiol. Gastrointest. Liver Physiol..

